# Immunogenic Profiling Reveals Promising RV-Identified Antigens as Vaccine Candidates Against *Klebsiella pneumoniae*

**DOI:** 10.3390/ijms27146398

**Published:** 2026-07-18

**Authors:** Ana Tajuelo, Eva Gato, Leilani Vaughan, Beatriz Cano-Castaño, Sonia Prieto Martín-Gil, Pedro Miguela-Villoldo, Antonio J. Martín-Galiano, Michael J. McConnell, Astrid Pérez

**Affiliations:** 1 Intrahospital Infections Laboratory, National Centre for Microbiology, Instituto de Salud Carlos III (ISCIII), 28220 Madrid, Spain or atajuelo11@alumno.uned.es (A.T.); eva.gato@isciii.es (E.G.); mali.vaughan99@gmail.com (L.V.); beatriz.cano@isciii.es (B.C.-C.); sonia.prieto@isciii.es (S.P.M.-G.); pedro.miguela@isciii.es (P.M.-V.); astrid.perez@isciii.es (A.P.); 2 Programa de Doctorado en Ciencias Biomédicas y Salud Pública, IMIENS, Universidad Nacional de Educación a Distancia (UNED), 28015 Madrid, Spain; 3 Core Scientific and Technical Units, Instituto de Salud Carlos III (ISCIII), 28220 Madrid, Spain; mgaliano@isciii.es; 4 Department of Biological Sciences, University of Notre Dame, Notre Dame, IN 46556, USA

**Keywords:** *Klebsiella pneumoniae*, protein vaccine, immune response, B-cell epitopes, strain recognition

## Abstract

Multidrug-resistant *Klebsiella pneumoniae* is an increasing global threat, and the limited availability of new antibiotics highlights vaccination as a promising strategy for infection prevention. Although different vaccine candidates have been evaluated, none is currently approved, mainly due to capsular heterogeneity among strains. Protein-based vaccines may overcome this limitation by targeting conserved epitopes. In this study, we assessed the immunogenicity of five outer membrane proteins (ChiP, LamB, RafY, OmpW, PagP) previously selected by reverse vaccinology (RV), comparing them with the well-characterized antigens OmpA and OmpK36. Mice were immunized intramuscularly with three doses of purified recombinant proteins, and antibody responses were analyzed by ELISA. All antigens elicited high, booster-induced IgG levels, with PagP slightly being less immunogenic. Regarding IgG subclasses, IgG_1_ predominated, followed by IgG_2b_ and IgG_2c_. Cross-reactivity was evaluated against six *K. pneumoniae* strains representing different clonal groups, and recognition by sera from previously infected mice was also examined. OmpA, ChiP and LamB showed the broadest cross-reactivity, while OmpA and LamB were most strongly recognized after infection. Overall, OmpA, LamB, ChiP and RafY emerged as the most promising vaccine candidates, although further optimization, such as a multicomponent vaccine, may be required. This work also highlights the importance of experimentally validating RV-selected antigens, as computational predictions alone do not ensure immunogenicity or in vivo relevance.

## 1. Introduction

The emergence of multidrug-resistant bacteria is a growing global problem that is reducing the effectiveness and availability of therapeutic options [1,2]. In this context, one of the most problematic pathogens is *Klebsiella pneumoniae* [3,4]. This Gram-negative pathogen is found in the environment and also on the mucosa of humans and animals, where it forms part of their commensal microbiota [5]. However, many serotypes are pathogenic, especially in immunocompromised and hospitalized patients, where they cause nosocomial infections such as pneumonia, urinary tract or wound infections, all of which can lead to bacteremia [6,7].

Historically, *K. pneumoniae* strains have been classified into two main pathogenic phenotypes: classical *K. pneumoniae* (cKp) strains, commonly associated with hospital-acquired infections that frequently harbor multiple antibiotic resistance determinants, and hypervirulent *K. pneumoniae* (hvKp) strains, which possess enhanced virulence traits that enable them to cause severe community-acquired infections, even in otherwise healthy individuals [8,9]. However, in recent years, strains combining both multidrug resistance and hypervirulence have been increasingly reported [10,11,12]. This convergence has led to an emergent reclassification of these specific strains as ultravirulent and supervirulent isolates, both of which have high virulence and resistance to multiple antibiotics, with the latter being lethal [13,14]. These strains represent a growing clinical concern, as they integrate the high pathogenic potential of hvKp with the limited therapeutic options associated with multidrug resistance.

The most common treatment for infections caused by *K. pneumoniae* has traditionally been β-lactam antibiotics; however, the prevalence of strains resistant to these compounds as well as to other broad spectrum antibiotics, such as carbapenems and fluoroquinolones, is steadily increasing [15,16]. In fact, carbapenem-resistant and third-generation cephalosporin-resistant *K. pneumoniae* have been designated critical priority pathogens by the World Health Organization (WHO) [17]. Given the limited availability of effective antibiotics, vaccine development is a promising alternative for preventing infections, and can also help to preserve the remaining active antibiotics by reducing their use [18,19]. However, to date there are no vaccines available against *K. pneumoniae*. Multiple strategies have been investigated for the development of a vaccine, such as attenuated bacteria, capsular polysaccharides, lipopolysaccharide (LPS) and their conjugates, among others [20,21,22,23]. However, most of these approaches have shown limitations with respect to the immune response generated and protection, with the main obstacle being the heterogeneity of the capsule and the lipopolysaccharide [24,25,26]. Sequence-based typing approaches have indicated that there are as many as 22 O-antigen types, and up to 167 K-antigen types [27]. This antigenic variability represents a critical challenge for the development of broadly active vaccines. In this context, highly conserved outer membrane proteins are an attractive alternative as they have potential to elicit antibodies that are protective across serotypes [28].

In the recent years, advances in computational biology have facilitated the application of reverse vaccinology (RV) principles to identifying potential proteins for vaccine development [29,30]. RV leverages in silico approaches to identify highly conserved proteins, predict B- and T-cell epitopes and also evaluate their potential immunogenicity and safety. RV is thus a powerful tool for identifying optimal candidates to be used as vaccine candidates, as it represents a more rational strategy than traditional vaccine development which has traditionally relied on empirical screening of antigens through complex, expensive and time consuming experiments [31]. Multiple studies have employed RV methods to identify potential vaccine candidates for *K. pneumoniae* [31,32,33,34]. However, relatively few of these studies have systematically validated these computationally identified antigens through experimental approaches. There is thus a critical gap in knowledge with regarding how computationally identified protein antigens perform as vaccine candidates in experimental models.

In a previous study by our group [35], we identified outer membrane proteins of *K. pneumoniae* as promising candidates for vaccine development using computational approaches. From this study, we have selected five proteins for in vivo experimental characterization: the sugar porins ChiP, LamB and RafY; the outer membrane protein OmpW, implicated in conjugation; and the lipid A palmoyltransferase PagP. We also included the outer membrane proteins OmpA and OmpK36 as controls, as previous studies demonstrated that immunization with these proteins elicited antigen-specific antibody responses in mouse models of *K. pneumoniae* and other Gram-negative bacteria [36,37,38,39]. These specific proteins were prioritized based on several key criteria previously validated in our group [35]: (i) extracellular location; (ii) high conservation across the six principal high-risk *K. pneumoniae* clonal groups (including ST11, ST15, ST37, ST147, ST307, and ST258); (iii) no potential cross-reactivity with human microbiota; and (iv) constitutive expression with an important function in bacterial physiology. Based on the high conservation established in our previous large-scale genomic analysis [35], the aim of the present study was to experimentally validate these reverse vaccinology-selected antigens by evaluating the humoral immune response they elicited in mouse, thereby assessing their potential as vaccine antigens.

## 2. Results

### 2.1. In Silico Characterization of the Selected Antigens

Five *K. pneumoniae* proteins—ChiP, LamB, OmpW, RafY and PagP—were selected as vaccine candidates by RV after applying multiple in silico analyses [35]. Porins OmpA and OmpK36 were also included in this work as controls, as they have been demonstrated to be immunogenic in previous studies with *K. pneumoniae* [36,37]. As summarized in Table 1, all are outer membrane proteins with a β-barrel structure ranging from 19 to 49 kDa. Most of them are implicated in nutrient transport but also contributing to pathogenesis and virulence. All proteins are predicted to have a moderately good solubility after removing the signal peptide, according to SoluProt 1.0. The five new candidates selected for this study are not predicted to be toxic or allergenic, based on ToxinPred2 and AllerCatPro 2.0., respectively, in contrast to OmpK36 and OmpA, which show weak evidence of being toxic (0.75) and allergenic, respectively.

### 2.2. Identification of Linear B-Cell Epitopes in the Selected Antigens

The number and surface exposure of B-cell epitopes in each protein were predicted (Table 2). OmpK36 was the protein with the highest number of predicted epitopic regions, with a total of eight, followed by LamB, RafY and OmpA, each with seven strong B-cell epitopes. All epitopes predicted for OmpK36, LamB and RafY are located on exposed regions of the β-barrel and have reasonable lengths to elicit an immune response. However only four of the epitopes predicted for OmpA, named in this work as OmpA_1, OmpA_2, OmpA_3 and OmpA_4, are in fact exposed, being part of four extracellular loops in the 3D structure. Regarding ChiP, six epitopic regions were predicted to be exposed, whereas PagP had five B-cell epitopes, of which PagP_2 is not exposed and only PagP_1 presents an optimal size for immune recognition. OmpW was the protein with the fewest epitopic regions, with only four, of which only three, OmpW_1, OmpW_3 and OmpW_4, are exposed, being OmpW_3 the only one long enough to potentially trigger an immune response.

### 2.3. Construction of Heterologous Expression Vectors and Purification of the Recombinant Proteins

**Figure 1:**
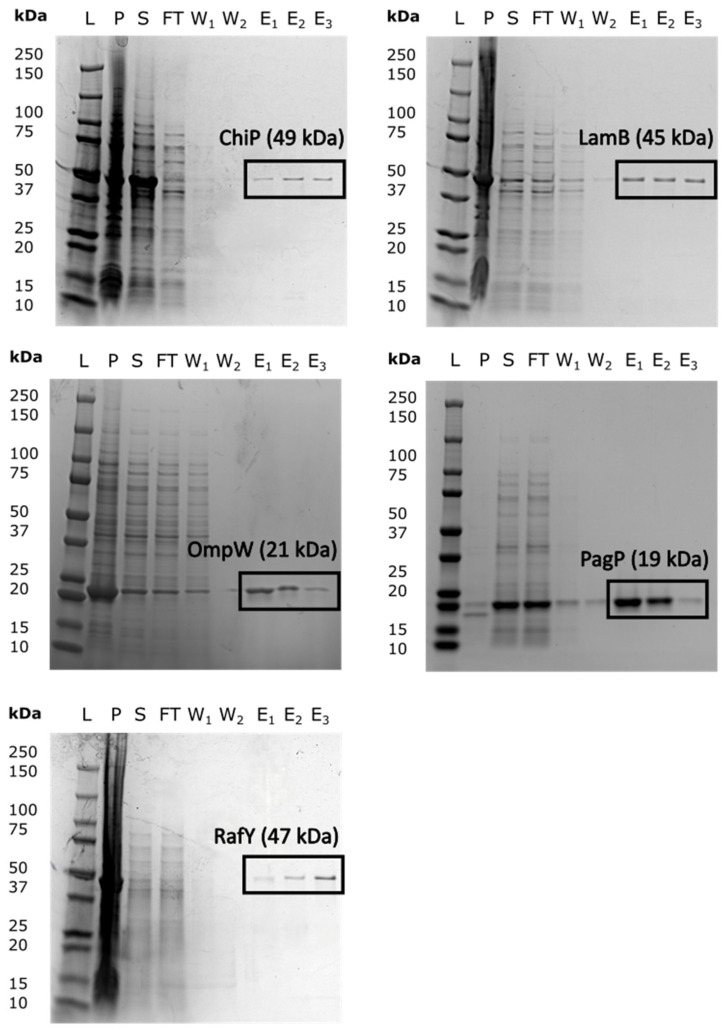
Protein purification with Ni-NTA columns. Each fraction was mixed with 2× Laemmli sample buffer supplemented with β-mercaptoethanol. Protein were resolved by SDS-PAGE and visualized by Coomassie staining. L: ladder; P: pellet fraction; S: supernatant fraction; FT: flow through fraction; W_1_, W_2_: washing fractions; E_1_, E_2_, E_3_: elution fractions.

The genes corresponding to the five selected proteins were highly expressed and purified using Ni-NTA affinity chromatography. To prevent protein accumulation in inclusion bodies, urea was used during purification. The purification process of the proteins was examined by SDS-PAGE, revealing a specific band at the expected size for each protein in the final elution fractions after washing steps (Figure 1).

### 2.4. RV-Identified Antigens Induce a Robust Humoral Response in Mice

**Figure 2:**
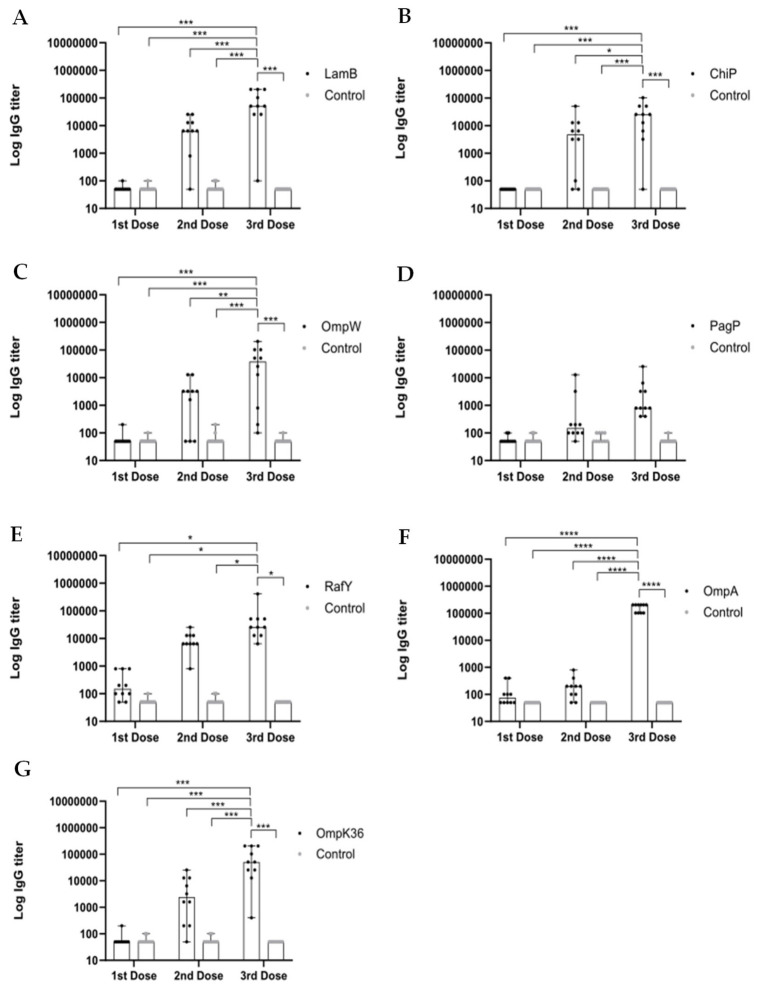
Post-immunization sera IgG titers. Specific IgG levels were quantified by ELISA at three distinct time points corresponding to each immunization dose for antigens (**A**–**G**). Groups of ten mice were immunized with either the recombinant protein formulated with adjuvant (black circles) or adjuvant alone (negative control, grey circles). Each data point represents an individual mouse, with horizontal lines and error bars indicating the median and range for each group. Statistical significance was assessed using a Kruskal–Wallis test followed by Dunn’s multiple comparison test; significance levels are denoted as follows: * *p* < 0.05, ** *p* < 0.001, *** *p* < 0.0001 and **** *p* < 0.00001. The Y-axis is presented on a log_10_ scale to reflect the magnitude of the humoral immune response.

To evaluate the immune response elicited by the seven recombinant proteins (ChiP, LamB, OmpW, RafY, PagP, OmpA and OmpK36), groups of mice were intramuscularly immunized with three doses of each purified recombinant protein on days 1, 15 and 29. Serum samples collected on days 14, 28 and 36 were used to evaluate the dynamic features of the immune response (Figure 2). For all the proteins, IgG titers remained almost undetectable after one dose and began to increase following the second immunization, reaching their highest levels after three doses, being significantly different to the control group. After the final dose, OmpA triggered the highest and most homogeneous response among mice, with titers ranging from 10^5^ and 2 × 10^5^ (Figure 2F). ChiP, LamB, OmpW and RafY also induced robust responses but slightly more variable among individual mouse, with titers ranging from 2 × 10^2^ to 2 × 10^5^ (Figure 2A, Figure 2B, Figure 2C and Figure 2E, respectively). In contrast, PagP was slightly less immunogenic, with titers ranging from 8 × 10^2^ to 2.6 × 10^4^ after three doses (Figure 2D). The control group of mice injected only with adjuvant did not exhibit any protein-specific antibody response throughout the immunization schedule, as expected.

**Figure 3:**
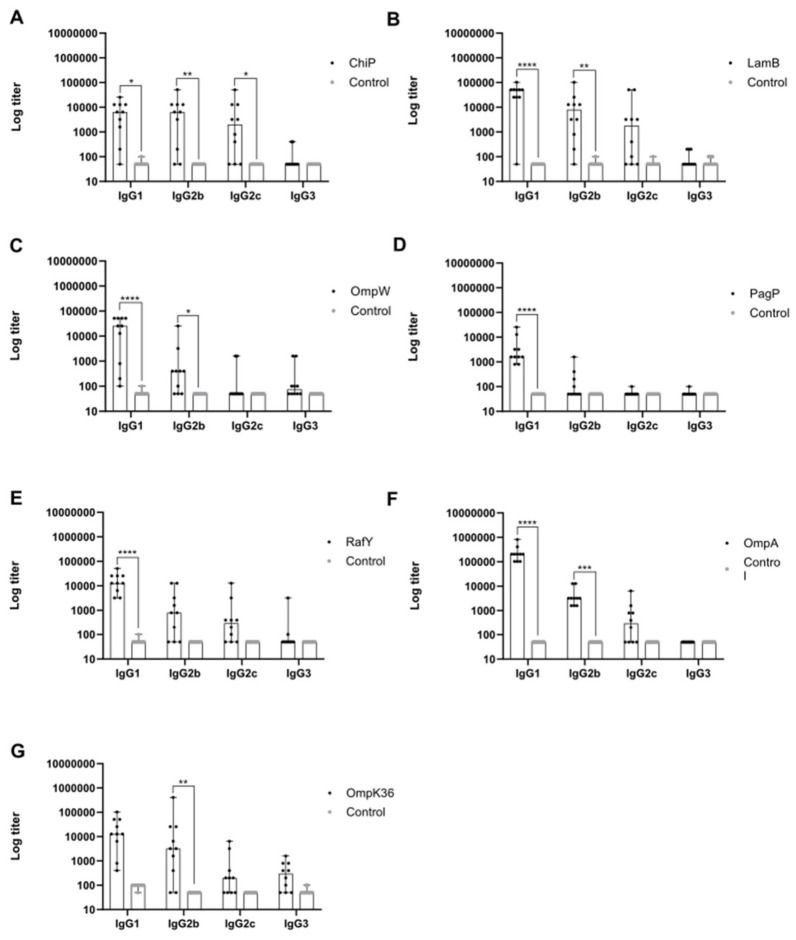
Sera IgG subclass titers. The distribution of specific IgG1, IgG2b, IgG2c, and IgG3 subclasses was quantified by ELISA for each target antigen (**A**–**G**) using serum samples collected after the third dose. Groups of 10 mice were immunized with either the recombinant protein formulated with adjuvant (black circles) or adjuvant alone (negative control, grey circles). Each data point represents an individual mouse, with horizontal lines and error bars indicating the median and range for each experimental group. Statistical significance was evaluated using a Kruskal–Wallis test followed by Dunn’s multiple comparison test; significance levels are indicated as follows: * *p* < 0.05, ** *p* < 0.001, *** *p* < 0.0001, and **** *p* < 0.00001. The Y-axis is presented on a log10 scale to reflect the magnitude of the humoral immune response.

Regarding IgG subclasses (Figure 3), after the three doses, the response was mostly dominated by IgG1 for all the antigens tested, reaching titers comparable to total IgG in most mice. ChiP, LamB, OmpW, OmpA and OmpK36 (Figure 3A, Figure 3B, Figure 3C, Figure 3F and Figure 3G, respectively) also induced strong IgG2b responses, with titers above 3200 in at least 60% of mice, while IgG2c levels were also high in most mice immunized with ChiP, reaching titers up to 5 × 10^4^ in some animals. In contrast, IgG3 was only detectable in few mice, especially those immunized with OmpW and OmpK36 (Figure 3E,G), but with titers not higher than 1.6 × 10^3^. IgM and IgA were also evaluated, but no detectable titers were observed.

### 2.5. Cross-Reactivity of Protein Specific Antibodies with Different K. pneumoniae Strains

**Figure 4:**
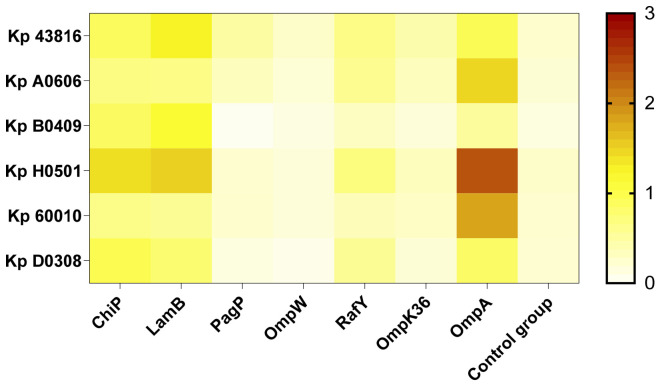
Cross-reactivity of mouse sera against *K. pneumoniae* strains measured by ELISA. The heatmap illustrates the immunoreactivity of pooled murine sera (collected after the final immunization dose and tested at a 1/100 dilution) against various *K. pneumoniae* clinical isolates. Antigens used for immunization are shown on the Y-axis, while the clinical strains categorized by their Sequence Type (ST) are listed along the X-axis. Color intensity represents the magnitude of the antigen-specific IgG interaction, with the numerical values in the color scale corresponding to the observed Optical Density (OD_450_) values. Each cell in the matrix reflects the mean OD_450_ obtained from two independent experimental assays. Pooled sera from mice receiving only adjuvant were used as a negative control to establish the baseline for color intensity and to ensure the specificity of the observed cross-reactivity.

Strain cross-reactivity of the immune sera was determined by ELISA against six *K. pneumoniae* strains belonging to different clonal groups (Table 3). As shown in the heatmap represented in Figure 4, the OmpA immune sera type was the most reactive, with a strong IgG response against all the strains tested, especially *K. pneumoniae* H0501, *K. pneumoniae* G0010 and *K. pneumoniae* A0606 (OD_450_ = 2.4, 1.8 and 1.5, respectively). Sera from mice immunized with ChiP, LamB and RafY also recognized all strains tested, showing their strongest reactivity toward *K. pneumoniae* H0501 (OD_450_= 1.4, 1.5 and 0.7, respectively) but with moderately lower IgG levels for the remaining strains. In contrast, sera from mice immunized with PagP, OmpW and OmpK36 barely reacted with any of the strains tested, reaching OD_450_ levels comparable to those of the negative control.

### 2.6. Protein Recognition by Sera from Infected Mice

**Figure 5:**
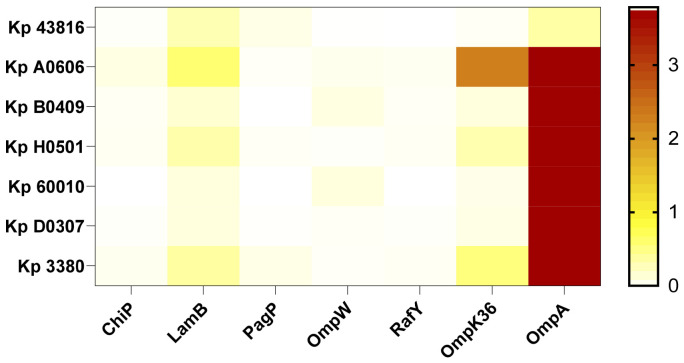
Recognition of recombinant proteins by sera from *K. pneumoniae*-infected mice. The heatmap displays the immunoreactivity of pooled sera collected from mice previously infected with different *K. pneumoniae* clinical strains, tested at a 1/100 dilution against the purified recombinant proteins. The X-axis identifies the target recombinant antigens, while the Y-axis lists the specific *K. pneumoniae* strains used for the primary infection. Color intensity directly reflects the magnitude of the antibody–antigen interaction, with the numerical scale representing the quantified Optical Density (OD_450_) values. Each individual cell in the matrix represents the mean OD_450_ calculated from two independent experimental assays. This visualization serves to confirm the natural exposure and subsequent recognition of these antigens during the course of a bacterial infection, using non-infected mouse sera as a baseline to define the specificity of the signals.

The immunoreactivity of IgG present in sera from infected mice with *K. pneumoniae* against the seven proteins was also evaluated (Figure 5). OmpA was strongly recognized by sera from mice infected with the six MDR strains tested, with OD_450_ values above 3. However, a marked reduction in the recognition was observed with sera from mice infected with the hypervirulent strain *K. pneumoniae* 43816 (OD_450_ = 0.4). OmpK36 and, to a lesser degree, LamB were well recognized by sera from mice infected with *K. pneumoniae* A0606 (OD_450_ = 2.3 and 0.6, respectively), and some recognition was also observed with sera from mice infected with *K. pneumoniae* H0501 and *K. pneumoniae* 3380, with OD_450_ values ranging from 0.4 to 0.6. LamB was also recognized to a limited extent following infection with *K. pneumoniae* 43816 (OD_450_ = 0.3). Low reactivity was observed against OmpW after infection with *K. pneumoniae* B0409 and *K. pneumoniae* G0010 (OD_450_ = 0.1), and ChiP was similarly recognized by sera from mice infected with *K. pneumoniae* A0606. Minimal reactivity was detected against PagP or RafY after infection with any of the strains.

## 3. Discussion

*K. pneumoniae* is one of the main pathogens responsible for nosocomial infections. Given the increasing number of MDR strains and the difficulty of finding new antimicrobials to combat them, vaccines represent a promising alternative to manage the infections [18,19,49]. However, there are no vaccines currently available against *K. pneumoniae*, although different attempts have been made [50]. In recent years, RV approaches have been applied to identify potential vaccine antigens in *K. pneumoniae* and other Gram-negative pathogens but a major limitation of many RV-based studies is the lack of subsequent experimental validation after computational prediction.

In the present study, we aimed to experimentally evaluate the immunogenicity of five outer membrane proteins of *K. pneumoniae* (ChiP, LamB, OmpW, RafY and PagP), previously selected by our group using RV [35], to assess their potential as vaccine candidates in a murine immunization model, comparing them with the well-characterized antigens OmpA and OmpK36, which have been tested before [36,37,38,39]. The in silico characterization determined that all are outer membrane proteins with a β-barrel structure, which is commonly associated with vaccine antigens candidates due to their accessibility and exposure to the immune system [51,52]. In addition, the lack of predicted toxicity or allergenicity for the five new candidates represents an advantage over OmpA and OmpK36, which showed weak evidence of toxicity or allergenicity (Table 1).

To further characterize the immunogenic potential of the selected proteins, we predicted the number and surface exposure of B-cell epitopes using BepiPred 3.0 software in combination with the three-dimensional structural model of the proteins. OmpK36 displayed the highest number of predicted epitopes, with eight regions, all exposed on the β-barrel and of sufficient length to potentially elicit an immune response, since they are greater than 10 amino acids [53]. OmpA contained seven predicted epitopes, but structural analysis revealed that only four are actually surface-exposed, as previously described [54]. All five additional proteins selected for this work presented exposed epitopic regions, especially LamB and RafY that have seven each, followed by ChiP with six potential B-cell epitopes. In contrast, PagP and OmpW present only one potential good epitope in terms of length and exposure, which could reduce the immune response.

The mapping of predicted linear B-cell epitopes onto AlphaFold 3 models allowed us to visualize the location of potentially immunogenic regions, mainly within the extracellular loops of the protein candidates. However, acknowledging the limitations of in silico linear predictions is essential, as the translation of these findings into a protective immune response is influenced by complex factors. In the context of outer membrane proteins with β-barrel architectures, a significant portion of the protective response may be directed toward conformational epitopes, which are not captured with this approach. This limitation is particularly relevant considering that all recombinant proteins were purified under denaturing conditions in this study to avoid aggregation and facilitate purification, which may have altered their native structure and affected the preservation of conformational epitopes. Therefore, the antibody responses observed in this study may predominantly reflect recognition of the linear epitopes. Although whole-cell ELISA results indicate that at least some antibodies recognize antigens on the bacterial surface, future studies evaluating protein refolding and structural recovery will be necessary to better define the contribution of conformational epitopes to immunogenicity and protection.

Furthermore, the relevance of these predictions depends on the epitopic density and length of these regions; proteins with longer and more frequent epitopes in their surface-exposed loops may exhibit higher accessibility. Additionally, factors such as immunodominance and the relative abundance of the antigen on the bacterial surface may play a decisive role in the overall IgG titers. Therefore, while our in silico mapping identifies candidate regions for immune recognition, and our results confirm that these proteins are immunogenic and accessible (as demonstrated by whole-cell ELISA), further experimental studies, such as peptide-based ELISAs, will be necessary to precisely define the contribution of each determinant to the protective landscape.

In order to experimentally confirm the immunogenicity of these antigens, the recombinant proteins were successfully expressed in *Escherichia coli* and purified by affinity chromatography under denaturing conditions to prevent protein aggregation. Mice immunization with three doses of 2 µg of the seven recombinant proteins induced antigen-specific IgG responses, with the highest titers after the final dose, although marked differences were observed in the magnitude and consistency of the responses. Our control protein OmpA elicited the highest and most homogeneous antibody titers, confirming its strong immunogenicity previously reported in *K. pneumoniae* and other Gram-negative pathogens [37,38,39,55,56]. Notably, ChiP, LamB, RafY and OmpW also induced robust humoral responses, but with greater inter-individual variability, which may reflect differences in epitope accessibility or antigen processing [57]. In contrast, PagP was clearly less immunogenic, indicating that, despite being selected as a promising candidate by *in silico* analysis, it elicited a weak response. This may be due to the lack of enough exposed epitopes, as we predicted before. However, this correlation is not absolute, since OmpW also had a single predicted exposed epitope but induced a stronger response, suggesting that other factors, like folding or expression level, may influence immunogenicity. Analysis of IgG subclasses (Figure 3) revealed a predominant IgG_1_ response after immunization with all the antigens, consistent with a predominant Th2 response [58]. However, ChiP, LamB, OmpW, OmpA and OmpK36 also induced substantial IgG_2b_ and IgG_2c_ responses in a significant proportion of mice. This is particularly relevant for protection against *K. pneumoniae*, as IgG_2_ subclasses, together with IgG_3_, are associated with enhanced opsonophagocytic activity and complement activation, both critical mechanisms for the clearance of encapsulated bacteria [59,60,61]. The low IgG_3_ levels may be explained by the fact that this subclass is predominantly induced by T-independent antigens, such as LPS, and is less efficiently generated in response to T-dependent antigens like proteins [62,63]. The limited detection of IgA after complete vaccination is likely related to the intramuscular immunization, since this antibody is mainly stimulated in the mucosa following intranasal immunization [64].

The assessment of strain cross-reactivity of the immune sera revealed marked differences among the antigens. OmpA immune sera showed the strongest and broadest reactivity against all tested *K. pneumoniae* strains. This finding is in agreement with the high conservation of this protein described across clonal lineages [65]. Moreover, OmpA has been shown to interact with antigen-presenting cells, inducing their maturation and deliver antigens into the MHC I pathway, supporting its ability to elicit humoral and also cellular responses, which makes it a strong antigen candidate [66]. Notably, sera from mice immunized with ChiP, LamB and RafY also recognized all strains tested, although the intensity of recognition was more variable. Despite the recombinant proteins being purified under denaturing conditions, the ability of these sera to bind intact bacterial cells indicates that immunization elicited antibodies capable of recognizing epitopes that remain accessible on the native bacterial surface. Although this observation does not distinguish between recognition of linear and conformational epitopes, it demonstrates that at least a subset of the antibody response is directed against naturally exposed regions. The variability observed among strains may reflect differences in antigen expression levels or regulation under specific growth condition and partial sequence divergence. Nevertheless, the ability of these sera to recognize multiple strains suggests a degree of antigenic conservation that could support cross-protective immunity with different *K. pneumoniae* strains. In contrast, PagP, OmpW and OmpK36 elicited antibodies with minimal or no reactivity against the tested strains, which may be explained by higher sequence variability, differential expression among strains or, as mentioned before, the limited surface exposed epitopes, at least under the experimental conditions tested in this work. For instance, OmpK36 is known to exhibit significant sequence diversity and is frequently altered or downregulated in MDR *K. pneumoniae* isolates, often as an adaptive response to antibiotic pressure [45,67,68]. Moreover, previous immunization studies using OmpK36 have also reported limited cross-protection with different species despite sequence similarity [69], highlighting that sequence conservation alone, often emphasized in RV studies, does not guarantee broad immunogenicity or antibody recognition in complex systems. Similarly, OmpW showed limited and inconsistent reactivity, which may be explained by condition-dependent expression or limited accessibility, as previously described in other *Enterobacteriaceae* like *E. coli* [70,71], which may reduce effective antibody binding despite immunogenicity in vitro. In addition, immunization with denatured OmpW may preferentially induce antibodies against linear epitopes that are not optimally exposed or accessible in the native conformation of the protein. Importantly, the reduced cross-reactivity observed for these antigens does not exclude their potential inclusion in a multivalent vaccine formulation, where antigen combination may broaden the overall immune coverage and compensate for individual variability in antigen recognition.

Evaluation of antigen recognition by sera from previously infected mice provided further insight into the biological relevance and in vivo availability of the selected proteins during a natural infection. OmpA was strongly recognized by sera from mice infected with all MDR strains tested, reinforcing its role as an immunodominant antigen expressed in vivo. However, its reduced recognition following infection with the hv strain suggests that antigen expression or accessibility may be altered in this background, potentially due to the presence of a thick hypermucoid capsule, a defined feature of hv *K. pneumoniae* that can limit the antibody access [72,73]. LamB and OmpK36 showed intermediate and strain-dependent recognition, indicating variability in their expression during infection. LamB is known to be regulated by nutrient availability and environment conditions [74], which may differ substantially between in vitro conditions and real host environment. Similarly, as mentioned before, *ompK36* expression is highly variable, and that can directly affect antigen accessibility to antibodies and can explain the inconsistent antibody responses observed following the infections. OmpW and ChiP displayed low reactivity with only minimal recognition by sera from specific infections, suggesting also limited exposure or weak immunogenicity. Similarly, the lack of detectable antibody responses against PagP and RafY suggests that these proteins may be poorly expressed or not sufficiently exposed during a systemic infection. These findings highlight the limitations of relying solely on in silico predictions without experimental confirmation, as not all computationally predicted candidates exhibit adequate immunogenicity, cross-reactivity or in vivo relevance.

Taken together, our results corroborate the strong immunogenicity of the antigen OmpA and identify LamB, ChiP and RafY as particularly promising vaccine antigens, as they combine all, or at least several, of the key desirable attributes, including robust immunogenicity, a favorable IgG subclass profile, notable good strain cross-reactivity and potential recognition during infection caused by some *K. pneumoniae* strains. These results should be considered as the experimental prioritization of vaccine antigens rather than evidence of protective efficacy. Further studies will be required to determine whether the immune responses elicited by these proteins translate into protection against infection, including antibody functional assays such as opsonophagocytic killing (OPKA) and serum bactericidal activity (SBA), characterization of antigen-specific cellular immune responses (e.g., T-cell activation and cytokine profiling) and in vivo challenge models. In addition, long-term immunization studies will be necessary to evaluate the durability of the antibody response, the establishment of immunological memory and the persistence of protective immunity.

In this context, a promising strategy for future research would be the evaluation of this antigens as a multicomponent formulation, combining the four antigens in the same vaccine. The optimization of a multicomponent formulation should consider a more detailed analysis of the antigenic regions responsible for the observed immune responses. In particular, the identification of conserved, surface-exposed and antibody-accessible B-cell epitopes, together with the prediction and experimental validation of T-cell epitopes, would provide valuable criteria for selecting the most informative antigen combinations. This approach could guide the development of whole-protein mixtures or, alternatively, rationally designed multiepitope constructs based on soluble scaffolds or other antigen-display platforms. Such strategies may help to focus the immune response, reduce potential antigenic competition and promote the induction of both broadly cross-reactive antibodies and antigen-specific cellular responses. Future work should therefore compare different antigen combinations and formulations, optimizing protein or epitope ratios, structural stability and adjuvant compatibility, while assessing functional antibody activity, T-cell activation and long-term immunological memory.

These approaches should be interpreted within the broader context of modern vaccine development strategies, which increasingly include diverse platforms such as mRNA-based vaccines, outer membrane vesicle (OMV) vaccines, and rationally designed recombinant subunit vaccines. In particular, the successful implementation of reverse vaccinology to the development of licensed vaccines, such as the *Neisseria meningitidis* serogroup B vaccine, highlights the value of combining computational antigen discovery with experimental validation [75,76].

## 4. Materials and Methods

### 4.1. Bacterial Strains, Plasmid and Culture Conditions

All bacterial strains and the plasmid used in the present work, along with the corresponding assays in which they were employed, are listed in Table 3. The bacterial strains used in this study were selected to represent the major high-risk clones of *K. pneumoniae*, which represent the most epidemiologically relevant and challenging threats currently found in clinical settings. These lineages (ST11, ST15, ST37, ST147, ST307, and ST258) are internationally recognized as ‘problem clones’ due to their global dissemination and the convergence of multidrug resistance and virulence factors, as established in the population genomics framework by Wyres et al. [6].

*K. pneumoniae* strains were cultured in Luria–Bertani (LB) (Thermo Fisher Scientific, Waltham, MA, USA) broth, whereas *E. coli* BL21 (D3) transformed with pET-21a plasmid was grown in LB supplemented with 100 µg/mL ampicillin. Strains were routinely grown at 37 °C with shaking, and for long-term storage, they were kept in LB containing 20% glycerol (*v*/*v*) and stored at −80 °C.

### 4.2. Selected Protein Characteristics

Proteins ChiP, LamB, OmpW, RafY and PagP (NCBI Protein accession numbers WIM74531.1, WP_202389409.1, YP_005226448.1, KGT66816.1 and HCA7088143.1, respectively) were selected to evaluate their immunogenicity from the previous RV study performed by our group [35]. In that work, these antigens were prioritized through extensive computational screening, which confirmed their high conservation across the major *K. pneumoniae* clonal groups, alongside other key vaccine candidate criteria. Proteins OmpA and OmpK36, routinely purified by our group, were also included as positive controls. Among the determinations carried out, subcellular location was predicted using PSORTb 3.0.3 (https://www.psort.org/psortb/, accessed on 7 July 2026). The 3D structures were obtained with AlphaFold 3 (https://alphafold.ebi.ac.uk/, accessed on 7 July 2026). Solubility was determined with SoluProt 1.0 (https://loschmidt.chemi.muni.cz/soluprot/, accessed on 7 July 2026), toxicity was evaluated with ToxinPred2 2.0 (https://webs.iiitd.edu.in/raghava/toxinpred2/batch.html, accessed on 7 July 2026) and allergenicity with AllerCatPro 2.0 (https://allercatpro.bii.a-star.edu.sg/, accessed on 7 July 2026).

### 4.3. Linear B-Cell Epitopes Prediction

Potential linear B-cell epitopes were predicted for each protein using BepiPred 3.0. The resulting epitopic sequences were subsequently mapped onto the 3D protein structures, generated by AlphaFold 3, in order to determine their localization and surface accessibility. Predicted epitopes were evaluated according to their exposure and their length to determine their suitability for effective immune recognition.

### 4.4. Construction of Expression Plasmids

The five selected proteins were cloned without the signal peptide (detected with SignalP 6.0 software, https://services.healthtech.dtu.dk/services/SignalP-6.0/, accessed on 7 July 2026) in the plasmid pET-21a (Novagen, Madison, WI, USA), which includes a 6xHis tag for the following purification. PCR amplification was performed from the genome of *K. pneumoniae* MGH78578 using the custom-designed primers and PCR conditions described in Table 4 and Table 5. PCR products were then digested with the correspondent restriction enzymes and ligated with the T4 ligase (174M0202T, New England Biolabs, Ipswich, MA, USA) into pET-21a vector.

### 4.5. Induction of Proteins

*E. coli* BL21 (D3) (C2527H, New England Biolabs, Ipswich, MA, USA) was transformed with the cloned plasmid pET-21a with the corresponding gene protein. A 10 mL culture of LB medium containing 100 μg/mL ampicillin was inoculated with a colony of recombinant *E. coli* BL21 (D3) and grown overnight at 37 °C with shaking at 150 rpm. One-hundred microliters of the overnight culture were used to inoculate fresh 500 mL of LB containing 100 μg/mL ampicillin. The culture was incubated at 37 °C with shaking until the OD_600_ reached 0.6 and then induced by the addition of 1 mM of isopropyl-b-D-thiogalactopyranoside (IPTG) (MB02603, NZYTech, Lisbon, Portugal) over 4 h. Cultures were then centrifuged at 4800 rpm for 15 min, and pellets were stored at −20 °C until protein purification.

### 4.6. Protein Purification Under Denaturing Conditions

For protein purification by affinity chromatography, the induced bacterial pellets were lysed with 10 mL of Lysis Buffer (100 mM NaH_2_PO_4_, 10 mM Tris-HCl, 8 M urea and 20 mM imidazole, pH = 8) for 1 h at room temperature (RT). Lysed samples were then centrifuged at 4800 rpm for 30 min at RT. The supernatant was added to a column containing 500 µL of Ni-NTA agarose (30210, Qiagen, Hilden, Germany), previously equilibrated in Lysis Buffer. Unbound proteins were washed twice using 4 mL of Washing Buffer (100 mM NaH_2_PO_4_, 10 mM Tris-HCl, 8 M urea, pH = 6.3). Finally, our protein was collected from the column by adding three fractions of 1 mL of Elution Buffer (100 mM NaH_2_PO_4_, 10 mM Tris-HCl, 4 M urea, pH = 4.5) and analyzed on 12% SDS-PAGE, where protein bands were visualized by Coomassie staining (Thermo Fisher Scientific, Walthman, MA, USA). Proteins were concentrated using ultra centrifugal filter of 10 kDa (AMICON^®^) (Merck Millipore, Burlington, MA, USA), and the final protein concentration was measured using the Qubit 4 Fluorometer (Invitrogen, Waltham, MA, USA) following the manufacturer’s instructions, using Qubit Protein Assay standards for calibration. All recombinant proteins were adjusted to a final concentration of 80 µg/mL prior to immunization. Endotoxin testing was not performed in this study; however, recombinant proteins were purified under denaturing and stringent washing conditions to minimize potential endotoxin contamination, and all immunizations were carried out using equal amounts of protein across experimental groups to ensure comparability between immunizations.

### 4.7. Mouse Immunization

Seven groups of 10 female mice C57BL/6 (5–6 weeks old, Charles Rivers, Saint Germain-Nuelles, France) were intramuscularly injected into the quadriceps muscle with three doses of 2 µg of each purified recombinant protein (ChiP, LamB, OmpW, RafY, PagP, OmpA and OmpK36). Each dose was formulated with aluminum hydroxide adjuvant (Alhydrogel^®^ adjuvant 2%, 21645-51-2 InvivoGen, San Diego, CA, USA) at a 1:1 ratio and administered at a total volume of 50 µL at intervals of two weeks. Aluminum hydroxide was selected because of its well-established safety profile, extensive use in licensed human vaccines, and proven ability to enhance humoral immune responses against protein antigens. Another group were injected only with Elution Buffer plus adjuvant as a negative control. Blood samples were collected from the retro-orbital plexus 24 h prior to the second and third doses, and a final exsanguination was carried out one week after the last dose of the vaccine. For downstream analyses, sera from mice within each group were pooled to obtain a representative sample per experimental condition.

All animal procedures were approved by the Animal Experimentation Ethics Committee of the Instituto de Salud Carlos III under protocol number M2-PROEX 228.7/20, and conducted in accordance with relevant national and European Union directives on animal experimentation (Directive 2010/63/EU).

### 4.8. Enzyme-Linked Immunosorbent Assays

Titers of protein-specific antibodies in sera were determined using an enzyme-linked immunosorbent assay (ELISA). For that, 96 well plates (159018, Cultek Corning, NY, USA) were coated overnight with 100 ng per well of each purified recombinant protein in PBS (Corning, NY, USA). After incubation, unbound proteins were removed by washing with 200 μL of 0.1% Tween 20 (Sigma-Aldrich, St. Louis, MO, USA) in PBS (PBST) (all subsequent washes were performed in the same way). After that, wells were blocked by adding 200 μL of PBST supplemented with 5% skim milk (Thermo Fisher Scientific, Waltham, MA, USA) for 1 h and washed twice. Serial two-fold dilutions of sera from immunized mice (ranging from 1:100 to 1:204,800) were added and incubated at RT for 1 h. Sera from individual mice were used as biological replicates (*n* = 10 per immunization group). After three washing steps, 100 μL of horseradish peroxidase (HRP)-conjugated anti-mouse IgG (1/10,000 dilution; A9044, Sigma-Aldrich, St. Louis. MO, USA), anti-mouse IgM (1/10,000 dilution; A8786, Sigma-Aldrich, St. Louis. MO, USA), anti-mouse IgG_1_ (1/10,000 dilution; SAB3701171, Sigma-Aldrich, St. Louis. MO, USA), anti-mouse IgG_2b_ (1/10,000 dilution; M32407, Thermo Fisher, Waltham, MA, USA), anti-mouse IgG_2c_ (1/10,000 dilution; ab97255, Abcam. Cambridge, UK), anti-mouse IgG_3_ (1/10,000 dilution; SAB3701192, Sigma-Aldrich, St. Louis, MO, USA) or anti-mouse IgA (1/10,000 dilution; A90-103P, Bethyl laboratories, Montgomery, TX, USA) was added to wells and incubated for 1 h at RT. Following four washing steps, 100 μL of HRP substrate (54827-17-7, Sigma-Aldrich, St. Louis, MO, USA) was added to each well and incubated for 15 min at RT. The enzymatic reaction was stopped by adding 50 μL 1 N sulfuric acid (Sigma-Aldrich, St. Louis, MO, USA). Absorbance at 450 nm was measured using a Sunrise^TM^ microplate reader (Tecan, Männedorf, Switzerland), and antibody titers were defined as the highest serum dilution yielding an OD_450_ value at least 0.1 above background wells (wells without serum). This protocol was also assed to determine the interaction of the seven proteins with IgG present in pooled sera (1/100 dilution) obtained from mice previously infected with seven *K. pneumoniae* strains (Table 3).

Whole-cell ELISAs were also carried out to determine the interaction with different *K. pneumonia* strains (Table 3). These assays were performed following the protocol described above with some modifications. Plates were coated with 100 µL/well of an OD_450_ 0.5 culture of the *K. pneumoniae* strain in carbonate coating buffer (CB01100, Thermo Fisher Scientific, (Waltham, MA, USA) and incubated overnight at 37 °C. After washing, plates were blocked for 2 h, and pooled sera from each immunized group, diluted at 1/100, were incubated overnight at 4 °C. Finally, plates were incubated with anti-mouse IgG (1/10,000 dilution) and OD_450_ values were determined. The analyses were performed in duplicate.

### 4.9. Statistical Analysis

Statistical analyses and data plotting were performed using Prism 5 v.5.01 (GraphPad Software). Antibody titers (log-transformed) were expressed as the median and range for each experimental group. Statistical differences between groups were evaluated using the Kruskal–Wallis nonparametric test followed by Dunn’s multiple-comparison post hoc test; *p*-values ≤ 0.05 were considered statistically significant.

## Figures and Tables

**Table 1:** Summary of the main characteristics of the selected proteins.

Protein	Molecular Weight (kDa)	Description	3D Structure	Location	Solubility	Toxicity	Allergenicity
			AlphaFold	PSORTb3	SoluProt 1.0	ToxinPred2	AllerCatPro 2.0
ChiP	49	Chitoporin implicated in the uptake of chitosugars (sugars associated with bacterial cell walls) [40].	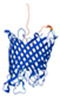	Outer membrane	0.718	Non-toxin (0.29)	No evidence
LamB	45	Maltoporin involved in the transportation of maltose and maltodextrins [41].	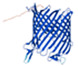	Outer membrane	0.839	Non-toxin (0.51)	No evidence
OmpW	21	Outer membrane protein with a role in conjugation that also contributes to virulence increasing resistance to host immune defense [42].	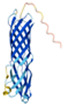	Outer membrane	0.588	Non-toxin (0.34)	No evidence
PagP	19	Lipid A palmoyltransferase that transfers palmitate from outer membrane phospholipids to lipid A [43].	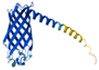	Outer membrane	0.826	Non-toxin (0.45)	No evidence
RafY	47	Glycoporin implicated in raffinose uptake [44].	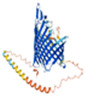	Outer membrane	0.664	Non-toxin (0.48)	No evidence
OmpK36	33	Outer membrane protein that contributes to nutrients uptake and decrease the antibiotic entry [45].	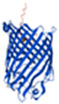	Outer membrane	0.864	Toxin (0.75)	No evidence
OmpA	40	Outer membrane protein implicated in adhesion and invasion to epithelial cell and macrophages and host immune evasion [46].	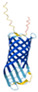	Outer membrane	0.829	Non-toxin (0.34)	Weak evidence

All predictions were carried out after removing the signal peptide detected with SignalP 6.0 software.

**Table 2:** Predicted B-cell epitopes for each protein.

Protein	Linear B-Cell Epitopes	Epitope Sequences
ChiP	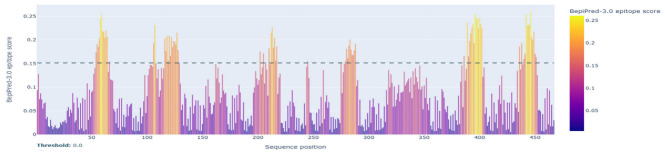	**ChiP_1:** KDVTDGDKYKT **ChiP_2:** GHPNEIAFSSRNKAYDEDYSGDK **ChiP_3:** WHIEMDDFYQNDKKTKV **ChiP_4:** KVSNGGVNDIYDGT **ChiP_5:** WDAKPGRMSSPDAYYDPDYRLKE **ChiP_6:** HSDIPSWSGGYGNIFQD
LamB	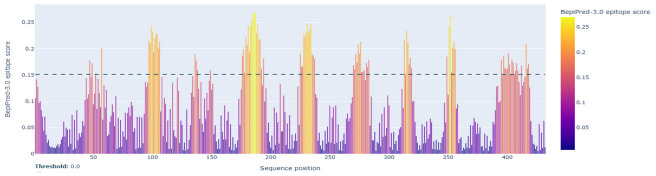	**LamB_1** QEDDWESTSPAF **LamB_2:** SGGSYTFSSDDTKKYAAK **LamB_3:** DDYRLEDGASKDG **LamB_4:** GHSQGTSIDNNGS **LamB_5:** DSKNGSTW **LamB_6:** SQRTSENN **LamB_7:** SNTSGLQTKDSSGSGAFTSSRGD
OmpW	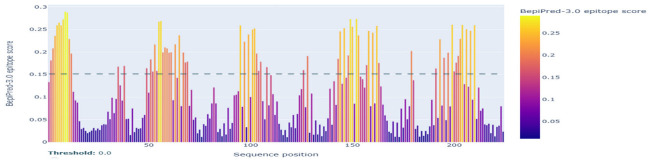	**OmpW_1:** NVLGSLGSF **OmpW_2:** ATVHQLPPT **OmpW_3:** EDFNDTGKAAGLSDLSLK **OmpW_4:** KAGGVD
PagP	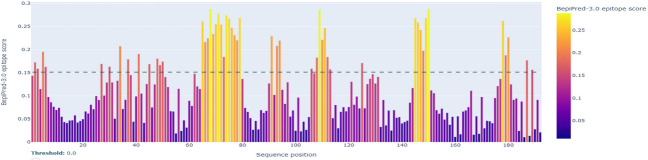	**PagP_1:** HARFAYDKEKTDKYNERPWGGA **PagP_2:** DEKGN **PagP_3:** KDSFNKWEP **PagP_4:** ARDNWNYI **PagP_5:** PGTYNNGN
RafY	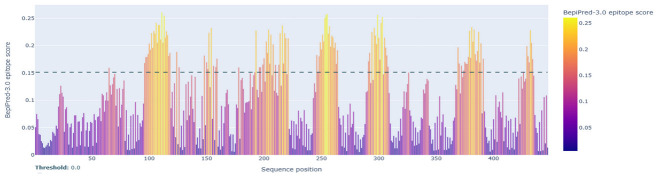	**RafY_1:** ASRTGSLTSVKTSANKSWAPGDKER **RafY_2:** LADMDGKMNLDD **RafY_3:**YSDGYGYIYMMKEGRG **RafY_4:** SSLFVDQNYHGNALENRKN **RafY_5:** NNAYGYQSQSGRWVDQSN **RafY_6:** DQFNMAGVTSECDGDCAILAPGR **RafY_7:** LDSTAAKSGNPD
OmpK36	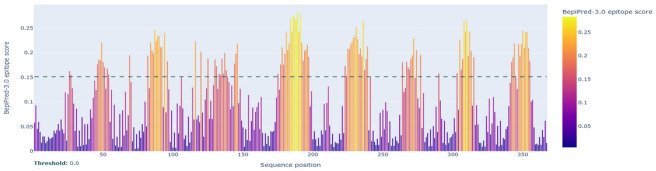	**OmpK36_1:** DDKSVDGDQ **OmpK36_2:** QANNTESSSDQAWTR **OmpK36_3:** GVVYDVTSWTDVLPEFGGDTYGSDNFLQSRAN **OmpK36_4:** QGKNGSVSGEGALSPTNNGRTALKQNG **OmpK36_5:** KRLGDQNSKLALGRGDN **OmpK36_6:** YNATRAGSLGFANK **OmpK36_7:** KDLEGYGDQDI **OmpK36_8:** LDDNSFTHNAGIST
OmpA	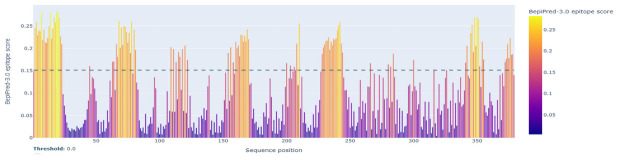	**OmpA_1:** SQYHDTGFYGNGFQNNNGPTRNDQ **OmpA_2:** LGRMAYKGSVDNGAFKA **OmpA_3:** DSKGNYASTGVSRSEH **OmpA_4:** QWVNNIGDAGTVGTRPDNGM **OmpA_5:** APVVAPAPAPAPEVA **OmpA_6:** GNTCDNVKARAALID **OmpA_7:** EVVTQPAA

Epitope sequences highlighted in green are surface-exposed, while those in red are non-exposed.

**Table 3:** Bacterial strains used and the experiment in which they were engaged.

Strain	Sequence Type	Phenotype	Assay	Reference
*E. coli* BL21 (D3)	ST93	Sensible	Protein expression	C2527H, New England Biolabs
*K. pneumoniae* MGH78578	ST52	MDR	Genomic DNA extraction for protein cloning procedure	ATCC
*K. pneumoniae* 43816	ST493	hv	ELISA (CR, ISR)	ATCC
*K. pneumoniae* A0606	ST307	MDR	ELISA (CR, ISR)	[47]
*K. pneumoniae* B0409	ST11	MDR	ELISA (CR, ISR)	[47]
*K. pneumoniae* H0501	ST147	MDR	ELISA (CR, ISR)	[47]
*K. pneumoniae* G0010	ST37	MDR	ELISA (CR, ISR)	[47]
*K. pneumoniae* D0308	ST147	MDR	ELISA (CR)	[47]
*K. pneumoniae* D0307	ST258	MDR	ELISA (ISR)	[47]
*K. pneumoniae* 3380	ST15	MDR	ELISA (ISR)	[48]
*Plasmid*	Assay			Reference
pET-21a	Protein cloning			Novagen

MDR: multidrug resistant; hv: hypervirulent. CR: cross-reactivity. ISR: infected serum recognition.

**Table 4:** Oligonucleotide primers used for protein cloning.

Gene	Primer Description	Sequence (5′ to 3′)
*chiP *	Chip forward (BamHI)	CGCGGATCCATGGCAGGCTTTATCGATGATTCA
	Chip reverse (HindIII)	CCCAAGCTTGAAAATAGTGAACGGGGCGAT
*lamB*	LamB forward (EcoRI)	CCGGAATTCATGGTCGATTTCCATGGCTACGCGCGTTCC
	LamB reverse (HindIII)	CCCAAGCTTCCACCACACTTCCATCTGGGCACCGAA
*ompW *	OmpW forward (EcoRI)	CCGGAATTCATGCATGAGGCGGGGGAGTTTTTC
	OmpW reverse (HindIII)	CCCAAGCTTGAACCGATAGCCTGCGGA
*rafY *	RafY forward (BamHI)	CGCGGATCCATGCAGGCGCCGTTATCG
	RafY reverse (HindIII)	CCCAAGCTTGAAGAAATATTTAAAGCGCACCCG
*pagP *	PagP forward (BamHI)	CGCGGATCCATGTCGTTTTCATCGACCCTTAGC
	PagP reverse (HindIII)	CCCAAGCTTAAACTGGATGCGCGC

Recognition sites of restriction enzymes are underlined, and the name of the enzyme is mentioned between parentheses.

**Table 5:** PCR conditions.

Step	Temperature	Time
Primary denaturation	95 °C	5 min
30 cycles		
Denaturation	95 °C	30 s
Annealing	62 °C	45 s
Elongation	72 °C	1 min/kb
Final extension	72 °C	7 min

PCR reactions were performed using Platinum Taq DNA Polymerase (Invitrogen, Thermo Fisher Scientific, USA) at a final reaction volume of 50 µL.

## Data Availability

The original contributions presented in this study are included in the article. Further inquiries can be directed to the corresponding author.

## Abbreviations

The following abbreviations are used in this manuscript:

RV	Reverse Vaccinology
*K. pneumoniae*	*Klebsiella pneumoniae*
ELISA	Enzyme-Linked Immunosorbent Assay
cKp	Classical Klebsiella pneumoniae
hvKp	Hypervirulent *Klebsiella pneumoniae*
WHO	World Health Organization
LPS	Lipopolysaccharide
kDa	Kilodalton
Ni-NTA	Nickel-Nitrilotriacetic Acid
SDS-PAGE	Sodium Dodecyl Sulfate–Polyacrylamide Gel Electrophoresis
OD	Optical Density
OPKA	Opsonophagocytic Killing Assay
SBA	Serum Bactericidal Assay
MHC I	Major Histocompatibility Complex class I
ST	Sequence Type
*E. coli*	*Escherichia coli*
LB	Luria–Bertani
MDR	Multidrug Resistant
Hv	Hypervirulent
PCR	Polymerase Chain Reaction
PBST	Phosphate-Buffered Saline with Tween 20
RT	Room Temperature
HRP	Horseradish Peroxidase
